# How parenting shapes the relationship between autistic traits and self-esteem in youth: a comparative study of autism spectrum disorder

**DOI:** 10.3389/fpsyt.2026.1747061

**Published:** 2026-02-26

**Authors:** Kazuhiko Yamamuro, Natsuko Kashida, Rio Ishida, Michihiro Toritsuka, Tsutomu Takeda, Manabu Makinodan

**Affiliations:** 1 Center for Health Control, Nara Medical University, Kashihara, Japan; 2 Department of Psychiatry, Nara Medical University School of Medicine, Kashihara, Japan; 3 Department of Psychiatry, Fujita Health University School of Medicine, Toyoake, Japan; 4 Division of Transformative Psychiatry and Synergistic Research, International Center for Brain Science (ICBS), Fujita Health University School of Medicine, Toyoake, Japan; 5 Department of Neuropsychiatry, Kumamoto University, Kumamoto, Japan

**Keywords:** adolescents, autism spectrum disorder, autistic traits, parenting style, self-esteem

## Abstract

**Background:**

Self-esteem is a critical factor in the psychological adjustment of children and adolescents, yet little is known about how autistic traits and parenting styles interact to relate self-worth in individuals with autism spectrum disorder (ASD). Understanding these relationships may provide important insights for family-based interventions.

**Methods:**

We conducted a cross-sectional study of 76 participants (ASD: n = 40; typically developing [TD]: n = 36). Autistic traits were assessed using the Japanese version of the Autism-Spectrum Quotient (AQ-J), parenting attitudes were evaluated with the Parental Nurturance and Parenting Scale (PNPS), and self-esteem was measured using the Rosenberg Self-Esteem Scale (RSES). In the ASD group, clinician-rated autism symptom severity was additionally assessed using the Autism Diagnostic Observation Schedule, Second Edition (ADOS-2). Associations among autistic traits, parenting attitudes, and self-esteem were examined using multiple linear regression and partial correlation analyses.

**Results:**

Children and adolescents with ASD exhibited significantly lower self-esteem than their TD peers (RSES: ASD < TD, p < 0.01). In the combined sample, higher AQ-J scores were independently associated with lower self-esteem (β ≈ −0.48, p < 0.01). Within the ASD group, negative parenting attitudes were linked to lower self-esteem (β = −0.36, p = 0.02), whereas positive parenting attitudes showed a non-significant trend toward higher self-esteem (β = 0.20, p = 0.17). Conversely, clinician-rated autism symptom severity assessed by the ADOS-2 was not associated with self-esteem (β = 0.06, p = 0.72). Overall, parenting attitudes were more closely related to self-esteem than clinician-rated symptom severity in the ASD group.

**Conclusion:**

These findings underscore the relevance of parenting contexts in relation to self-esteem among youth with ASD. Although autistic traits were associated with greater vulnerability in self-esteem, supportive parenting attitudes were associated with more favorable self-esteem outcomes. Together, the results suggest that parenting-related factors may be important considerations when addressing psychological well-being in autistic children and adolescents.

## 1 Introduction

Autism spectrum disorder (ASD) is a neurodevelopmental condition characterized by impairments in social communication, interaction, and restricted or repetitive behaviors (1). In this study, the term ASD is used in accordance with current diagnostic frameworks and established clinical research, while recognizing ongoing discussions within the autistic community regarding preferred terminology (2). Beyond these core features, individuals with ASD frequently experience elevated rates of anxiety and depression (3, 4), both of which are closely associated with self-esteem (5). Self-esteem, defined as an individual’s global sense of self-worth, is fundamental to mental well-being (6), and its reduction is recognized as a risk factor for depressive and anxious symptoms (7). Consistent evidence shows that autistic youth and adults report lower self-esteem than their typically developing (TD) peers (8, 9), highlighting a critical area of vulnerability that warrants systematic investigation.

Multiple factors contribute to challenges in self-esteem among individuals with ASD. Social difficulties are central, as impairments in initiating and maintaining friendships often result in isolation, rejection, and feelings of inadequacy (10). Approximately half of all youth with ASD are reported to lack close friendships (11), and prevalence studies consistently reveal elevated bullying rates in this population (12). One survey found that more than 70% of school-aged autistic children experienced bullying within a single month (13)—a rate significantly higher than in neurotypical peers or even in other disability groups. Such peer victimization undermines self-worth (14) and associates subsequent declines in mental health (15). Conversely, supportive peer relationships can buffer these effects; adolescents who feel included report less loneliness and higher self-esteem (11). Another important factor is heightened self-awareness of differences. As children mature, some autistic youth become more conscious of their divergence from peers (16). Theory of mind refers to the ability to attribute mental states, such as beliefs and intentions, to oneself and others. In autistic youth, more advanced theory-of-mind abilities may increase sensitivity to social comparison and peer evaluation, thereby heightening awareness of social exclusion, victimization, or interpersonal failure. As a consequence, heightened theory of mind can paradoxically be associated with lower self-esteem, because it facilitates more accurate and often self-critical interpretations of social experiences, particularly in contexts of peer rejection or bullying (8, 9). A persistent sense of “being different,” compounded by negative external feedback, can further erode confidence and self-image (17).

The family environment is strongly linked to the self-esteem of autistic children. Raising a child with ASD often places considerable strain on caregivers, who report higher levels of stress (18) and reduced feelings of parenting efficacy (19). These stressors may limit the availability of warmth, praise, or positive reinforcement, thereby hindering healthy self-concept development. Research has linked negative or controlling parenting styles have been linked to poorer outcomes in social skills, autonomy, and emotional adjustment (20). In contrast, nurturing family environments—characterized by warmth, encouragement, and recognition of strengths—can promote resilience and support the development of a positive self-image (21). Supportive parenting not only fosters emotional growth but may also protect against the adverse effects of external stressors, such as bullying or social rejection (17).

Intervention research highlights the malleability of self-esteem in ASD. Adaptations of cognitive-behavioral therapy have been piloted to address self-image concerns, with improvements in confidence and self-worth (7). Mindfulness-based group therapies for autistic youth have reduced anxiety and increased social confidence (18). Skills-based programs, such as those using magic-trick training, provide mastery experiences that indirectly build self-esteem (17). Peer mentoring and community-based initiatives that emphasize neurodiversity acceptance also show promise (21), with evidence that a positive autistic identity is linked to higher self-esteem and better mental health (22). Collectively, these findings suggest that although low self-esteem is prevalent in ASD, it is not immutable and can be improved through targeted interventions.

Despite growing attention, significant research gaps remain. Previous studies have often examined isolated factors, with few integrating autistic traits and family dynamics within a single framework (8, 15). Autistic traits refer to autism-related characteristics that are continuously distributed in the general population and are not limited to individuals with a clinical diagnosis. Moreover, most intervention studies remain preliminary or limited in scope (7). This study addresses these gaps by examining the associations between autistic traits and self-esteem in children and adolescents, with a particular focus on parenting attitudes. We hypothesized that higher levels of autistic traits would be associated with lower self-esteem, and that variations in parenting attitudes would be related to self-esteem outcomes. In addition, we explored whether these associations differed between autistic and TD youth. By clarifying these relationships, this study aims to contribute to a more nuanced understanding of factors associated with self-esteem vulnerability in ASD.

## 2 Methods

### 2.1 Participants

A total of 76 children and adolescents aged 9–15 years participated in the study, including 40 with a clinical diagnosis of ASD and 36 TD controls. Participants in the ASD group were recruited from patients receiving care at the Department of Psychiatry, Nara Medical University Hospital, between March 2021 and January 2024. Participants with ASD were recruited from an outpatient psychiatric service during the study period, with eligible patients enrolled upon providing informed consent. TD participants were recruited employing convenience sampling from the local community. Diagnoses of ASD were established by at least two experienced psychiatrists or clinical psychologists according to the criteria of the Diagnostic and Statistical Manual of Mental Disorders, 5th edition (DSM-5) (1). To ensure diagnostic accuracy, only individuals who also met ASD classification criteria on the Autism Diagnostic Observation Schedule, Second Edition (ADOS-2; Modules 3–4) were included. The ADOS-2 is widely recognized as the gold-standard observational assessment for ASD, with strong validity in differentiating autism from other developmental and psychiatric conditions (23).

Cognitive functioning was assessed using the Das–Naglieri Cognitive Assessment System (DN-CAS), a theory-driven instrument that evaluates four cognitive processing domains: planning, attention, simultaneous processing, and successive processing (24). Participants with a total DN-CAS score below 70 were excluded to minimize the influence of intellectual disability, consistent with previous studies that regard a score below 70 as indicative of significant cognitive impairment (24). Individuals with other neurological or psychiatric disorders were also excluded from the study.

Demographic information, including age and sex, was obtained from caregiver questionnaires and medical records. Written informed consent was obtained from all parents or legal guardians, and assent was obtained from the children when appropriate. The study protocol was approved by the Ethics Committee of Nara Medical University (Approval Nos. 1319, 2535).

### 2.2 Autism spectrum quotient–Japanese version

The Autism Spectrum Quotient–Japanese version (AQ-J) is a 50-item self-report questionnaire that evaluates autistic traits across five core domains: social skills, attention switching, attention to detail, communication, and imagination. Each item is rated on a 4-point Likert scale, with higher scores indicating greater levels of autistic traits. Example items include “I find it difficult to make new friends” (social skills), “I frequently get so absorbed in one thing that I lose sight of other things” (attention switching), and “I find it difficult to read between the lines in conversations” (communication). The AQ-J has been validated for use in Japanese populations and demonstrates good internal consistency (Cronbach’s α = 0.77–0.86) (25). Because all participants were under 16 years of age, assessments were completed by a parent or caregiver.

### 2.3 Rosenberg self-esteem scale

The Rosenberg Self-Esteem Scale (RSES) is a widely used 10-item measure designed to estimate global self-worth and self-esteem (26). It includes positively and negatively worded statements, such as “I take a positive attitude toward myself” and “I feel I do not have much to be proud of.” Each item is rated on a four-point Likert scale, ranging from “strongly disagree” to “strongly agree,” with higher scores showing greater self-esteem. The Japanese version, translated and validated by Yamamoto et al., has shown high internal consistency (Cronbach’s α = 0.81) and good construct validity (27, 28). Individuals with ASD often present with low self-esteem, making this measure especially useful for capturing clinically meaningful variation in self-worth within this population. The RSES, originally developed for adolescents and widely used in late childhood and adolescence, was considered appropriate for participants aged 9–15 years (26, 29). The scale was completed by the children themselves, with brief explanations provided to younger participants as needed; caregivers did not influence responses.

### 2.4 Parental nurturance profile scale

Parenting style was assessed using the Parental Nurturance Profile Scale (PNPS), a validated instrument that evaluates parental attitudes and behaviors across supportive and maladaptive domains. The PNPS consists of multiple subscales, including involvement, positive responsivity, respect for will, overprotection, inconsistency, harsh discipline, positive parenting, and negative parenting. Higher scores on positive domains (e.g., involvement, responsivity, respect for will) indicate greater parental warmth and support, whereas higher scores on negative domains (e.g., harsh discipline, overprotection, inconsistency) reflect more controlling or maladaptive parenting practices. The PNPS has been widely applied in Japanese child and adolescent research, where it has illustrated strong internal consistency, construct validity, and clinical utility in distinguishing parenting behaviors associated with children’s socio-emotional adjustment (30, 31). Although the PNPS was not developed specifically for ASD, it has been applied in clinical and developmental research including children with neurodevelopmental and emotional–behavioral difficulties. In this study, the PNPS was used to capture general dimensions of parenting attitudes relevant across diagnostic groups.

### 2.5 Statistical analysis

Analyses were conducted in the combined TD + ASD sample and separately in the ASD and TD groups to examine dimensional and group-specific associations among autistic traits, parenting attitudes, and self-esteem. Within the ASD group, parallel regression analyses using parent-reported autistic traits (AQ-J) and clinician-rated autism severity (ADOS-2) were performed to distinguish subjective trait expression from clinician-observed symptom severity.

All analyses were two-tailed with a significance level set at α = 0.05. Descriptive data are presented as mean ± standard deviation (SD). Group differences between individuals with ASD and TD individuals in continuous variables were examined using independent-samples t-tests, whereas sex distribution was compared using chi-squared (χ²) tests. Effect sizes are reported as Cohen’s d for continuous variables and Cramer’s V for categorical variables. The 95% confidence intervals refer to the mean differences between groups.

To examine associations between autistic traits, parenting attitudes, and self-esteem, linear regression analyses were first conducted. Self-esteem, assessed by the RSES total score, was entered as the dependent variable. Parent-reported autistic traits (AQ-J total score) and parenting attitudes (PNPS assessed by the parent) were entered as independent variables. In the pooled TD + ASD sample, age was included as a covariate in Model 1, and age and diagnostic status (ASD vs. TD) were included in Model 2. Additional regression analyses were conducted separately in the TD group and the ASD group, with age included as a covariate. In analyses restricted to the ASD group, clinician-rated autism symptom severity assessed by the ADOS-2 total score was examined alongside parenting attitudes to compare the relative associations of clinician-rated severity and parent-reported autistic traits with self-esteem.

Next, path analyses were conducted to examine the direct and indirect associations among autistic traits, parenting attitudes, and self-esteem using observed variables. Autistic traits (AQ-J total score), parenting attitudes, and self-esteem were modeled simultaneously, with parenting attitudes hypothesized to mediate the association between autistic traits and self-esteem. Parenting attitudes were modeled separately as positive and negative domains based on PNPS subscales. Analyses were performed for the pooled TD + ASD sample as well as separately for the TD and ASD subgroups. Given the cross-sectional design, these paths represent statistical associations rather than causal relationships. Two-tailed p values and 95% confidence intervals were calculated, and age was included as a covariate in all models.

Finally, partial correlation analyses were conducted as supplementary analyses to further characterize the associations among autistic traits, parenting attitudes, and self-esteem while controlling for potential confounding variables. In the pooled TD + ASD sample, partial correlations were calculated controlling for age and diagnostic status. In analyses restricted to the ASD group, partial correlations were calculated controlling for age only and included associations with ADOS-2 total scores. Partial correlation coefficients (r) and corresponding significance levels are reported in **Supplementary Tables 5, 6**.

## 2 Results

### 2.6 Participant characteristics

A total of 76 participants were included in the analysis (ASD group: n = 40; TD comparison group: n = 36). There were no significant group differences in age (*p* = 0.76, *d* = -0.07) or sex distribution (*p* = 0.71). Group comparisons of demographic variables, autism-related traits, self-esteem, and parenting style are summarized in **Table 1**. Regarding autism-related traits assessed by the AQ-J, the ASD group had significantly higher scores than the TD group in social skills (*p* < 0.01, *d* = -1.43), attention switching (*p* < 0.01, *d* = -1.27), local details (*p* = 0.02, *d* = -0.68), communication (*p* < 0.01, *d* = -1.62), imagination (*p* < 0.01, *d* = -0.94), and total AQ-J score (27.58 ± 8.08 vs. 14.30 ± 6.42; *p* < 0.01, *d* = -1.83). On the RSES, the ASD group reported significantly lower self-esteem than the TD group (*p* < 0.01, *d* = 0.94). For parenting style, a significant between-group difference was observed only for the PNPS subscale of involvement (*p* = 0.02, *d* = 0.74). No significant differences were observed on other PNPS subscales, including positive responsivity (*p* > 0.99, *d* = -0.05), respect for will (*p* > 0.99, *d* = 0.17), overprotection (*p* = 0.20, *d* = -0.53), inconsistency (*p* > 0.99, *d* = 0.07), harsh discipline (*p* = 1.00, *d* = -0.17), positive parenting (*p* > 0.99, *d* = 0.33), and negative parenting (*p* > 0.99, *d* = -0.32). Within the ASD group, symptom severity was evaluated using the ADOS-2. The mean scores were 3.5 ± 1.0 for the language and communication domain, 11.6 ± 2.9 for reciprocal social interaction, and 0.4 ± 0.8 for restricted and repetitive behaviors, with a mean total score of 12.1 ± 3.1. These findings confirm the presence of core autism-related impairments in communication and social reciprocity within the ASD group.

**Table 1:** Demographic characteristics, autistic traits, self-esteem, and parenting style in the ASD and TD groups.

	TD	ASD	*P*-value	Cohen’s D	95% confidence interval	
	N = 40	N = 36			Lower	Upper
Age (years)	11.18 (2.33)	11.33 (2.26)	0.76	-0.07	-0.52	0.38
Sex (male/female)	25/15	21/15	0.71			
AQ-J						
Social skill	0.35 (2.58)	6.83 (2.71)	**< 0.01**	-1.43	-1.43	-1.93
Attention switching	3.13 (1.74)	5.61 (2.18)	**< 0.01**	-1.27	-1.27	-1.76
Local details	3.23 (1.90)	4.58 (2.08)	**0.02**	-0.68	-0.68	-1.15
Communication	1.98 (1.89)	5.53 (2.50)	**< 0.01**	-1.62	-1.62	-2.13
Imagination	2.93 (2.22)	5.03 (2.24)	**< 0.01**	-0.94	-0.94	-1.42
Total score	14.30 (6.42)	27.58 (8.08)	**< 0.01**	-1.83	-1.83	-2.34
RSES						
Total score	28.85 (5.14)	23.58 (6.08)	**< 0.01**	0.94	0.94	0.46
PNPS						
Involvement	53.75 (9.78)	46.58 (9.67)	0.02	0.74	0.74	0.27
Positive responsivity	50.58 (10.50)	51.11 (9.34)	> 0.99	-0.05	-0.05	-0.50
Respect for will	52.48 (11.37)	50.53 (11.39)	> 0.99	0.17	0.17	-0.28
Overprotection	49.98 (9.39)	54.89 (9.27)	0.20	-0.53	-0.53	-0.98
Inconsistency	52.98 (11.48)	52.11 (12.56)	> 0.99	0.07	0.07	-0.38
Harsh discipline	50.88 (12.15)	52.72 (9.06)	> 0.99	-0.17	-0.17	-0.62
Positive Parenting	52.95 (11.70)	49.33 (10.16)	> 0.99	0.33	0.33	-0.13
Negative Parenting	51.58 (11.09)	54.92 (9.93)	> 0.99	-0.32	-0.32	-0.77
ADOS-2						
Language and communication	NA	3.5 (1.0)				
Reciprocal and interaction	NA	11.6 (2.9)				
Restricted and repetitive stereotyped behaviors and interests	NA	0.4 (0.8)				
Total score	NA	12.1 (3.1)				

Values are presented as mean (standard deviation) unless otherwise indicated. Group differences between the typically developing (TD) and autism spectrum disorder (ASD) groups were examined using independent-samples t-tests for continuous variables and a chi-square test for sex distribution. Effect sizes are reported as Cohen’s d. The 95% confidence intervals are provided for both the mean differences between groups and the corresponding Cohen’s d values. Bold values indicate statistical significance (p < 0.05).

TD, typically developing; ASD, autism spectrum disorder; AQ-J, Autism Spectrum Quotient, Japanese version; PNPS, Parental Nurturance Profile Scale; ADOS-2, Autism Diagnostic Observation Schedule, Second Edition; RSES, Rosenberg Self-Esteem Scale.

### 2.7 Associations of autistic traits and parenting attitudes with self-esteem

In the pooled sample including TD and ASD participants, linear regression analyses were conducted to examine the associations of parent-reported autistic traits and parenting attitudes with self-esteem (**Table 2**). In Model 1, which adjusted for age, higher levels of parent-reported autistic traits (AQ-J total score) were significantly associated with lower self-esteem (β = −0.471, p < 0.001). Neither positive parenting (β = 0.186, p = 0.104) nor negative parenting (β = −0.021, p = 0.858) showed a significant association with self-esteem. In Model 2, which additionally adjusted for diagnostic status (ASD vs. TD), the negative association between autistic traits and self-esteem remained significant (β = −0.371, p = 0.011). Parenting attitudes again did not show significant independent associations with self-esteem (positive parenting: β = 0.180, p = 0.116; negative parenting: β = −0.033, p = 0.776). These findings indicate that autistic traits were independently associated with self-esteem across the pooled sample, even after accounting for age and diagnostic group.

**Table 2:** Regression models examining associations of parenting attitudes and autistic traits with self-esteem in the pooled TD + ASD sample.

Dependent variable and covariate	B	SE	β	*t*-value	*p*-value	95% confidence interval		R^2^	Adjusted R^2^	*p*-value
						Lower	Upper			
RSES^1^								0.316	0.277	**< 0.001**
PNPS: Positive Parenting	0.103	0.063	0.186	1.648	0.104	-0.176	0.303			
PNPS: Negative Parenting	-0.012	0.067	-0.021	-0.180	0.858	-0.427	0.054			
AQ-J: Total by parent	-0.195	0.-066	-0.471	-4.475	**< 0.001**	-0.543	0.765			
RSES^2^								0.326	0.278	0.299
PNPS: Positive Parenting	0.100	0.063	0.180	1.592	0.116	-0.025	0.225			
PNPS: Negative Parenting	-0.019	0.067	-0.033	-0.286	0.776	-0.153	0.115			
AQ-J: Total by parent	-0.233	0.089	-0.371	-2.608	0.011	-0.410	-0.055			

Linear regression analyses were conducted with self-esteem (Rosenberg Self-Esteem Scale total score; RSES1) as the dependent variable and parent-reported autistic traits (AQ-J total) as well as parenting attitudes (positive and negative parenting assessed by the Parent–Nurturing Parenting Scale) as independent variables. Bold values indicate statistical significance (p < 0.05).

In Model 1, age was included as a covariate. In Model 2, age and diagnostic status (autism spectrum disorder [ASD] vs. typically developing [TD]) were included as covariates. Regression coefficients are presented as unstandardized coefficients (B), standard errors (SE), standardized coefficients (β), t-values, and p-values. R² and adjusted R² indicate the proportion of variance explained by each model. All analyses were conducted in the pooled sample including TD and ASD participants.

RSES, Rosenberg Self-Esteem Scale; AQ, Autism-Spectrum Quotient; PNPS, Parent–Nurturing Parenting Scale; ASD, autism spectrum disorder; TD, typically developing.

To examine whether these associations differed by diagnostic group, additional regression analyses were conducted separately within the TD group (**Supplementary Table 1**). After adjusting for age, parent-reported autistic traits were not significantly associated with self-esteem in TD participants (β = −0.087, p = 0.616). Similarly, neither positive parenting (β = 0.283, p = 0.122) nor negative parenting (β = 0.063, p = 0.733) showed a significant association with self-esteem in this group. Parallel analyses were conducted within the ASD group (**Supplementary Table 2**). In contrast to the TD group, higher levels of parent-reported autistic traits were significantly associated with lower self-esteem after adjusting for age (β = −0.442, p = 0.008). Positive parenting (β = 0.115, p = 0.519) and negative parenting (β = −0.171, p = 0.345) were not significantly associated with self-esteem.

### 2.8 Associations between autistic traits and parenting attitudes

In the pooled TD + ASD sample, parent-reported autistic traits were associated with parenting attitudes (**Table 3**). Specifically, higher autistic trait levels were associated with higher levels of negative parenting at the coefficient level after adjustment for age (Model 1: β = 0.32, p = 0.005). This association remained statistically significant after further adjustment for diagnostic status (ASD vs. TD) (Model 2: β = 0.40, p = 0.011), although the overall model fit did not reach statistical significance. In contrast, autistic traits were not significantly associated with positive parenting in either model, although a trend toward lower positive parenting was observed when controlling for age alone (Model 1: β = −0.23, p = 0.051).

**Table 3:** Regression models examining associations between parent-reported autistic traits and parenting attitudes in the pooled TD + ASD sample.

Dependent variable and covariate	B	SE	β	*t*-value	*p*-value	95% confidence interval		R^2^	Adjusted R^2^	*p*-value
						Lower	Upper			
PNPS: Positive Parenting^1^								0.065	0.040	0.085
AQ: Total by parent	-0.256	0.129	-0.228	-1.988	0.051	-0.514	0.001			
PNPS: Positive Parenting^2^								0.065	0.027	0.179
AQ: Total by parent	-0.247	0.178	-0.219	-1.389	0.169	-0.602	0.107			
PNPS: Negative Parenting^1^								0.105	0.081	**0.017**
AQ: Total by parent	0.347	0.121	0.321	2.868	**0.005**	0.106	0.589			
PNPS: Negative Parenting^2^								0.112	0.075	**0.035**
AQ-J: Total by parent	0.433	0.166	0.400	2.601	**0.011**	0.101	0.764			

Linear regression analyses examined associations between parent-reported autistic traits (AQ-J total) and parenting attitudes (positive and negative parenting) assessed by the Parent–Nurturing Parenting Scale (PNPS). Bold values indicate statistical significance (p < 0.05).

In Model 1, age was included as a covariate. In Model 2, age and diagnostic status (autism spectrum disorder [ASD] vs. typically developing [TD]) were included as covariates. Positive parenting and negative parenting were examined as dependent variables in separate regression models, with parent-reported AQ total entered as the independent variable. Standardized regression coefficients (β) are shown. Analyses were conducted in the pooled sample including TD and ASD participants.

RSES, Rosenberg Self-Esteem Scale; AQ, Autism-Spectrum Quotient; PNPS, Parent–Nurturing Parenting Scale; ASD, autism spectrum disorder; TD, typically developing.

When analyses were stratified by diagnostic group, no statistically significant associations were observed between autistic traits and parenting attitudes in either group. In the TD group (**Supplementary Table 3**), higher autistic traits showed a trend-level association with increased negative parenting (β = 0.31, p = 0.058), whereas the association with positive parenting was weak and non-significant (β = −0.20, p = 0.225). Similarly, in the ASD group (**Supplementary Table 4**), autistic traits were not significantly associated with parenting attitudes; however, a non-significant tendency toward greater negative parenting was observed (β = 0.29, p = 0.092), while no association was evident for positive parenting (β = −0.13, p = 0.457).

### 2.9 Associations of parenting attitudes and clinician-rated autism severity with self-esteem in the ASD Group

Within the ASD group, linear regression analyses were conducted to examine the associations of parenting attitudes and clinician-rated autism symptom severity with self-esteem (**Table 4**). Self-esteem (RSES total score) was entered as the dependent variable, and positive parenting, negative parenting, and ADOS-2 total scores were entered as independent variables, with age included as a covariate. Clinician-rated autism symptom severity, as assessed by the ADOS-2 total score, was not significantly associated with self-esteem (β = 0.057, p = 0.732). Similarly, neither positive parenting (β = 0.107, p = 0.591) nor negative parenting (β = −0.305, p = 0.123) showed a significant association with self-esteem in this model.

**Table 4:** Regression models examining associations between parenting attitudes, clinician-rated autism severity, and self-esteem in the ASD group.

Dependent variable and covariate	B	SE	β	*t*-value	*p*-value	95% confidence interval		R^2^	Adjusted R^2^	*p*-value
						Lower	Upper			
RSES^1^								0.149	0.039	0.273
PNPS: Positive Parenting	0.064	0.117	0.107	0.543	0.591	-0.176	0.303			
PNPS: Negative Parenting	-0.187	0.118	-0.305	-1.585	0.123	-0.427	0.054			
ADOS-2: Total	0.111	0.321	0.057	0.345	0.732	-0.543	0.765			

Linear regression analysis was conducted with self-esteem (Rosenberg Self-Esteem Scale total score; RSES) as the dependent variable. Parenting attitudes were assessed using the Parent–Nurturing Parenting Scale (PNPS), including positive and negative parenting. Clinician-rated autism severity was assessed using the Autism Diagnostic Observation Schedule, Second Edition (ADOS-2) total score. In Model 1, age was included as a covariate. Unstandardized regression coefficients (B), standard errors (SE), standardized coefficients (β), t-values, p-values, and 95% confidence intervals are reported. R² and adjusted R² indicate the proportion of variance explained by the model.

RSES, Rosenberg Self-Esteem Scale; PNPS, Parent–Nurturing Parenting Scale; ADOS-2, Autism Diagnostic Observation Schedule, Second Edition; ASD, autism spectrum disorder.

### 2.10 Partial correlations among autistic traits, parenting attitudes, and self-esteem

Partial correlation analyses in the pooled TD + ASD sample (**Supplementary Table 5**) provided complementary support for the regression findings. After controlling for age and diagnostic status, higher parent-reported autistic traits were moderately associated with lower self-esteem (r = −0.52, Bonferroni-adjusted p < 0.001). Autistic traits were also positively associated with negative parenting (r = 0.32, p < 0.05) and inversely associated with positive parenting, although the latter did not reach statistical significance after correction. In addition, positive parenting was positively correlated with self-esteem (r = 0.30, p < 0.05), whereas negative parenting showed a weak and non-significant inverse association with self-esteem after correction. These correlational patterns were consistent with the direction of effects observed in the regression analyses.

Within the ASD group (**Supplementary Table 6**), partial correlation analyses controlling for age indicated that higher parent-reported autistic traits were significantly associated with lower self-esteem (r = −0.50, Bonferroni-adjusted p < 0.05). In contrast, clinician-rated autism symptom severity (ADOS-2 total score) was not significantly correlated with self-esteem. Negative parenting showed a moderate, non-significant inverse association with self-esteem, whereas positive parenting was weakly and positively correlated with self-esteem; however, neither association remained statistically significant after Bonferroni correction. Autistic traits were moderately correlated with negative parenting, but this association did not reach adjusted significance.

## 3 Discussion

This study examined the relationships among autistic traits, parenting style, and self-esteem in children and adolescents with and without ASD. Consistent with prior research, higher levels of autistic traits were associated with lower self-esteem, particularly in the pooled sample and within the ASD group, highlighting vulnerability associated with autism-related characteristics. Although overall parenting styles did not differ substantially between participants with ASD and those with TD, variations in parenting attitudes were related to self-esteem at the correlational level. Negative parenting tendencies were associated with poorer self-esteem, whereas positive parenting tendencies showed modest associations in the opposite direction. In contrast, autism symptom severity as measured by the ADOS-2 was not independently associated with self-esteem after adjustment. Taken together, these findings suggest that self-esteem in autistic youth may be more closely related to subjective autistic traits and relational contexts than to clinician-rated symptom severity, underscoring the importance of considering family-related factors when interpreting psychosocial outcomes.

### 3.1 Autistic traits and self-esteem in youth

Children and adolescents with ASD exhibited significantly lower self-esteem than their TD (8, 32). Low self-esteem in autism is well documented and often co-occurs with depression and anxiety (9, 17). In both groups, self-esteem has been shown to be inversely associated with depressive symptoms (7), reinforcing its significance as a risk factor for mental health difficulties. Beyond group comparisons, higher levels of autistic traits were associated with lower self-esteem in the combined sample, consistent with evidence that even subclinical traits may undermine self-concept (33). In contrast, autism symptom severity as assessed by the ADOS-2 was not directly associated with self-esteem, suggesting that clinician-rated symptom severity alone may be less closely related to self-evaluative outcomes. Previous studies have indicated that factors such as alexithymia, rather than autism severity per se, are associated with low self-esteem in autistic adolescents (34). Taken together, these findings suggest that vulnerabilities in self-esteem may reflect not only autistic traits but also broader social-emotional characteristics and contextual responses. Given that poor self-esteem in ASD is associated with depression, anxiety, and suicidality (35, 36), identifying risk and protective factors remains an important clinical and research priority.

### 3.2 Parenting style in relation to autistic traits

Our findings showed no broad differences in overall parenting style between the ASD and TD groups, consistent with meta-analytic evidence indicating comparable levels of parental warmth and support across diagnostic groups (37). However, higher levels of autistic traits were associated with more negative parenting attitudes and showed modest associations with lower positive parenting, echoing prior reports that parents of children with ASD may exhibit slightly greater control or negativity (38, 39). These associations may reflect the increased stress and challenges associated with managing communication difficulties or behavioral characteristics (19, 40). Importantly, substantial variability was observed within each group, and many parents of children with ASD reported highly supportive parenting attitudes. Taken together, these findings suggest that differences in parenting attitudes may be less strongly related to diagnostic status per se than to individual stress levels, coping strategies, and contextual supports (37–39).

### 3.3 Parenting influences on children’s self-esteem

Parenting style was related to self-esteem outcomes at the correlational level. Tendencies toward negative parenting—such as harshness, inconsistency, or overprotection—were associated with poorer self-esteem, with a more pronounced pattern observed in youth with ASD (42). Previous research has shown that harsh parenting is linked to greater emotional and behavioral difficulties in children (43), which may in turn undermine self-evaluative processes. In contrast, positive parenting characterized by warmth, acceptance, and support for autonomy showed modest associations with higher self-esteem, consistent with findings in neurotypical and clinical populations (41, 42). Positive parenting has also been associated with reduced vulnerability to stress and adversity (22), suggesting that supportive parenting contexts may be relevant to self-esteem development in autistic youth. Although subgroup analyses did not consistently yield statistically significant results, the overall pattern of associations across groups was directionally consistent.

### 3.4 Transactional perspectives on parenting and self-esteem

Parenting processes are often conceptualized within transactional frameworks, in which child characteristics and parenting behaviors are thought to influence one another over time. Although cross-sectional data cannot establish causality, such models highlight the potential for bidirectional associations between child difficulties and parental responses (44). In this context, our findings are broadly consistent with a transactional perspective, in that higher autistic traits tended to co-occur with elevated negative parenting tendencies, which were in turn associated with lower self-esteem (43). Conversely, positive parenting attitudes were modestly associated with higher self-esteem, suggesting that supportive relational contexts may be relevant to children’s self-evaluative processes. The absence of a direct association between clinician-rated autism severity (ADOS-2) and self-esteem further suggests that relational and contextual factors may be more closely linked to self-esteem than symptom severity alone. Other factors, such as social support and self-regulation, may also play important roles in shaping self-esteem (17).

### 3.5 Clinical and practical implications

Promoting self-esteem in children with ASD may be an important clinical consideration, given its broad associations with mental health and overall well-being (6). Prior intervention studies suggest that parent-focused programs aimed at enhancing warmth, praise, and autonomy support can improve parent–child interactions (45), and meta-analytic evidence indicates benefits for a range of ASD-related outcomes (46). Because parenting practices are modifiable, supportive parenting contexts may be relevant targets for psychosocial support. In addition, parental well-being warrants attention, as higher parental stress has been associated with harsher parenting practices (40, 47). Mindfulness-based programs have been shown to reduce parental stress and reactivity (47), highlighting the potential value of approaches that address child and parent factors.

### 3.6 Limitations and future directions

This study is limited by its cross-sectional design, reliance on parent- and self-reported measures, and modest sample size. Longitudinal research is needed to clarify the temporal ordering and potential causal directions among autistic traits, parenting attitudes, and self-esteem (48). Accordingly, all associations identified in the present analyses should be interpreted as correlational rather than causal. Future studies would benefit from incorporating observational and multi-informant approaches, as well as larger and more diverse samples. In addition, peer relationships and experiences such as bullying warrant further consideration, given their established associations with self-esteem (15, 49). Cross-cultural replication will also be important to evaluate the generalizability of these findings.

## 4 Conclusion

Autistic traits were associated with lower self-esteem, and parenting style was related to this association at the correlational level. Negative parenting tendencies were linked to greater self-esteem difficulties, whereas positive parenting tendencies showed associations in the opposite direction. In contrast, autism symptom severity itself was not directly associated with self-esteem, suggesting that relational and contextual factors may be more closely related to self-evaluative outcomes than clinician-rated symptom severity. Together, these findings highlight the potential relevance of supportive parenting contexts when considering psychosocial well-being in autistic youth.

## Data Availability

The raw data supporting the conclusions of this article will be made available by the authors, without undue reservation.
