# Supplementary material for: How parenting shapes the relationship between autistic traits and self-esteem in youth: a comparative study of autism spectrum disorder
Source: Front Psychiatry. 2026 Feb 26;17:1747061. doi: 10.3389/fpsyt.2026.1747061 (PMC12979555; doi:10.3389/fpsyt.2026.1747061)
Supplement: Supplementary file 1 [file Table1.docx]

**Supplementary Table 1. Regression models examining associations with self-esteem in the TD group.**

| **Dependent variable and covariate** |  |  |  |  |  | **95% confidence interval** | | |  |  |  |
| --- | --- | --- | --- | --- | --- | --- | --- | --- | --- | --- | --- |
|  | **B** | **SE** | **β** | ***t*-value** | ***p*-value** | **Lower** |  | **Upper** | **R^2^** | **Adjusted R^2^** | ***p*-value** |
| RSES^1^ |  |  |  |  |  |  |  |  | 0.100 | -0.003 | 0.434 |
| PNPS: Positive Parenting | 0.124 | 0.078 | 0.283 | 1.585 | 0.122 | -0.035 |  | 0.283 |  |  |  |
| PNPS: Negative Parenting | 0.029 | 0.085 | 0.063 | 0.344 | 0.733 | -0.143 |  | 0.202 |  |  |  |
| AQ-J: Total by parent | -0.070 | 0.138 | -0.087 | -0.507 | 0.616 | -0.350 |  | 0.210 |  |  |  |

Linear regression analyses were conducted with self-esteem (Rosenberg Self-Esteem Scale total score; RSES1) as the dependent variable in participants with typical development (TD) only. Parent-reported autistic traits (AQ-J total) and parenting attitudes (positive and negative parenting assessed by the Parent–Nurturing Parenting Scale) were entered as independent variables. In Model 1, age was included as a covariate. Regression coefficients are presented as unstandardized coefficients (B), standard errors (SE), standardized coefficients (β), t-values, and p-values. R² and adjusted R² indicate the proportion of variance explained by the model.

**Abbreviations:** RSES, Rosenberg Self-Esteem Scale; AQ-J, Autism-Spectrum Quotient, Japanese version; PNPS, Parent–Nurturing Parenting Scale; TD, typically developing

**Supplementary Table 2. Regression models examining associations with self-esteem in the ASD group**

| **Dependent variable and covariate** |  |  |  |  |  | **95% confidence interval** | | |  |  |  |
| --- | --- | --- | --- | --- | --- | --- | --- | --- | --- | --- | --- |
|  | **B** | **SE** | **β** | ***t*-value** | ***p*-value** | **Lower** |  | **Upper** | **R^2^** | **Adjusted R^2^** | ***p*-value** |
| RSES^1^ |  |  |  |  |  |  |  |  | 0.319 | 0.232 | **0.015** |
| PNPS: Positive Parenting | 0.069 | 0.105 | 0.115 | 0.653 | 0.519 | -0.146 |  | 0.283 |  |  |  |
| PNPS: Negative Parenting | -0.105 | 0.109 | -0.171 | -0.960 | 0.345 | -0.327 |  | 0.118 |  |  |  |
| AQ-J: Total by parent | -0.332 | 0.118 | -0.442 | -2.816 | **0.008** | -0.573 |  | -0.092 |  |  |  |

Linear regression analyses were conducted with self-esteem (Rosenberg Self-Esteem Scale total score; RSES1) as the dependent variable in participants with autism spectrum disorder (ASD) only. Parent-reported autistic traits (AQ-J total) and parenting attitudes (positive and negative parenting assessed by the Parent–Nurturing Parenting Scale) were entered as independent variables. In Model 1, age was included as a covariate. Regression coefficients are presented as unstandardized coefficients (B), standard errors (SE), standardized coefficients (β), t-values, and p-values. R² and adjusted R² indicate the proportion of variance explained by the model.

**Abbreviations:** RSES, Rosenberg Self-Esteem Scale; AQ-J, Autism-Spectrum Quotient, Japanese version; PNPS, Parent–Nurturing Parenting Scale; ASD, autism spectrum disorder

**Supplementary Table 3. Regression models examining associations between parent-reported autistic traits and parenting attitudes in the TD group**

| **Dependent variable and covariate** |  |  |  |  |  | **95% confidence interval** | | |  |  |  |
| --- | --- | --- | --- | --- | --- | --- | --- | --- | --- | --- | --- |
|  | **B** | **SE** | **β** | ***t*-value** | ***p*-value** | **Lower** |  | **Upper** | **R^2^** | **Adjusted R^2^** | ***p*-value** |
| PNPS: Positive Parenting^1^ |  |  |  |  |  |  |  |  | 0.041 | -0.011 | 0.461 |
| AQ: Total by parent | -0.369 | 0.299 | -0.202 | -1.233 | 0.225 | -0.974 |  | 0.237 |  |  |  |
| PNPS: Negative Parenting^1^ |  |  |  |  |  |  |  |  | 0.095 | 0.046 | 0.159 |
| AQ: Total by parent | 0.539 | 0.275 | 0.312 | 1.956 | 0.058 | -0.019 |  | 1.097 |  |  |  |

Linear regression analyses were conducted to examine associations between parent-reported autistic traits (AQ-J total score) and parenting attitudes assessed by the Parent–Nurturing Parenting Scale (PNPS) in participants with typical development (TD) only. Positive parenting and negative parenting were examined as dependent variables in separate regression models, with parent-reported AQ-J total score entered as the independent variable. Age was included as a covariate in all models. Regression coefficients are presented as unstandardized coefficients (B), standard errors (SE), standardized coefficients (β), t-values, p-values, and 95% confidence intervals. R² and adjusted R² indicate the proportion of variance explained by each model.

**Abbreviations:** AQ-J, Autism-Spectrum Quotient, Japanese version; PNPS, Parent–Nurturing Parenting Scale; TD, typically developing

**Supplementary Table 4. Regression models examining associations between parent-reported autistic traits and parenting attitudes in the ASD group**

| **Dependent variable and covariate** |  |  |  |  |  | **95% confidence interval** | | |  |  |  |
| --- | --- | --- | --- | --- | --- | --- | --- | --- | --- | --- | --- |
|  | **B** | **SE** | **β** | ***t*-value** | ***p*-value** | **Lower** |  | **Upper** | **R^2^** | **Adjusted R^2^** | ***p*-value** |
| PNPS: Positive Parenting^1^ |  |  |  |  |  |  |  |  | 0.065 | 0.008 | 0.331 |
| AQ: Total by parent | -0.161 | 0.214 | -0.128 | -0.753 | 0.457 | -0.598 |  | 0.275 |  |  |  |
| PNPS: Negative Parenting^1^ |  |  |  |  |  |  |  |  | 0.093 | 0.038 | 0.200 |
| AQ: Total by parent | 0.358 | 0.206 | 0.291 | 1.735 | 0.092 | -0.062 |  | 0.778 |  |  |  |

Linear regression analyses were conducted to examine associations between parent-reported autistic traits (AQ-J total score) and parenting attitudes assessed by the Parent–Nurturing Parenting Scale (PNPS) in participants with autism spectrum disorder (ASD) only. Positive parenting and negative parenting were examined as dependent variables in separate regression models, with parent-reported AQ-J total score entered as the independent variable. Age was included as a covariate in all models. Regression coefficients are presented as unstandardized coefficients (B), standard errors (SE), standardized coefficients (β), t-values, p-values, and 95% confidence intervals. R² and adjusted R² indicate the proportion of variance explained by each model.

**Abbreviations:** AQ-J, Autism-Spectrum Quotient, Japanese version; PNPS, Parent–Nurturing Parenting Scale; ASD, autism spectrum disorder

**Supplementary Table 5. Partial correlations among autistic traits, parenting attitudes, and self-esteem in the pooled TD + ASD sample**

| Variables | AQ-J total | PNPS Positive | PNPS Negative | RSES |
| --- | --- | --- | --- | --- |
| AQ-J total | **-** | -0.227 | **0.318*** | **-0.518***** |
| PNPS Positive |  | **-** | **-0.473***** | **0.302*** |
| PNPS Negative |  |  | **-** | -0.258 |
| RSES |  |  |  | **-** |

Partial correlation coefficients (r) are shown for associations among parent-reported autistic traits (AQ-J total score), parenting attitudes assessed by the Parent–Nurturing Parenting Scale (PNPS; Positive Parenting and Negative Parenting), and self-esteem assessed by the Rosenberg Self-Esteem Scale (RSES). Partial correlations were calculated controlling for age and diagnostic status (autism spectrum disorder [ASD] vs. typically developing [TD]). To account for multiple comparisons, Bonferroni correction was applied by multiplying the original p values by six. Statistical significance is indicated using Bonferroni-adjusted p values as p < 0.05 (*), p < 0.01 (**), and p < 0.001 (***).

**Abbreviations:** AQ-J, Autism-Spectrum Quotient, Japanese version; PNPS, Parent–Nurturing Parenting Scale; RSES, Rosenberg Self-Esteem Scale; ASD, autism spectrum disorder; TD, typically developing.

**Supplementary Table 6. Partial correlations among autistic traits, parenting attitudes, and self-esteem in the ASD group**

| Variables | AQ-J total | ADOS-2 | PNPS Positive | PNPS Negative | RSES |
| --- | --- | --- | --- | --- | --- |
| AQ-J total | **-** | -0.158 | -0.130 | 0.289 | **-0.503*** |
| ADOS-2 |  | **-** | -0.044 | 0.064 | 0.034 |
| PNPS Positive |  |  | **-** | **-0.502*** | 0.256 |
| PNPS Negative |  |  |  | **-** | -0.355 |
| RSES |  |  |  |  | **-** |

Partial correlation coefficients (r) are shown for associations among parent-reported autistic traits (AQ-J total score), clinician-rated autism symptom severity assessed by the Autism Diagnostic Observation Schedule, Second Edition (ADOS-2 total score), parenting attitudes assessed by the Parent–Nurturing Parenting Scale (PNPS; Positive Parenting and Negative Parenting), and self-esteem assessed by the Rosenberg Self-Esteem Scale (RSES). Partial correlations were calculated controlling for age. Positive values indicate positive associations, and negative values indicate inverse associations. To account for multiple comparisons, Bonferroni correction was applied by multiplying the original p values by ten. Statistical significance is indicated using Bonferroni-adjusted p values as p < 0.05 (*), p < 0.01 (**), and p < 0.001 (***)

**Abbreviations:** AQ-J, Autism-Spectrum Quotient, Japanese version; ADOS-2, Autism Diagnostic Observation Schedule, Second Edition; PNPS, Parent–Nurturing Parenting Scale; RSES, Rosenberg Self-Esteem Scale; ASD, autism spectrum disorder.
